# A Fasting‐Mimicking Diet Affects the Inflammatory Response Following Periodontal Treatment: A Multi‐centre Feasibility Randomised Controlled Pilot Trial

**DOI:** 10.1111/jcpe.70139

**Published:** 2026-06-10

**Authors:** Giuseppe Mainas, Elena Figuero, Marta Amigo Basilio, José Dopico, Florencia Julieta Gayo Morales, Antonio Magan‐Fernandez, Inmaculada Cabello, Guillermo Pardo Zamora, Josefina Guillén Sanchez, Jose Nart, Antonio Santos Alemany, Carlos Pereira Couto, Manlio Vinciguerra, Valter D. Longo, Mark Ide, Mariano Sanz, Luigi Nibali

**Affiliations:** ^1^ Periodontology Unit, Centre for Host Microbiome Interactions, Faculty of Dentistry, Oral & Craniofacial Sciences King's College London London UK; ^2^ ETEP (Etiology and Therapy of Periodontal and Peri‐Implant Diseases) Research Group, Department of Dental Clinical Specialties, Faculty of Dentistry University Complutense of Madrid Madrid Spain; ^3^ Postgraduate Programme of Periodontology, Department of Dental Clinical Specialties Complutense University of Madrid Madrid Spain; ^4^ Periodontology Unit, Faculty of Odontology University of Santiago de Compostela Santiago de Compostela Spain; ^5^ Periodontology Unit, Department of Stomatology, School of Dentistry University of Granada Granada Spain; ^6^ Department of General Dentistry and Implants, Faculty of Medicine and Dentistry University of Murcia Murcia Spain; ^7^ Department of Periodontology Universitat Internacional de Catalunya Barcelona Spain; ^8^ Department of Translational Stem Cell Biology, Research Institute Medical University of Varna Varna Bulgaria; ^9^ Department of Medicine and Surgery LUM University Casamassima Italy; ^10^ Longevity Institute, Leonard Davis School of Gerontology, Department of Biological Sciences University of Southern California Los Angeles California USA

**Keywords:** diet, fasting, gingival crevicular fluid, inflammation, periodontitis

## Abstract

**Aim:**

To investigate whether cycles of fasting‐mimicking diet (FMD), used as an adjunct to non‐surgical periodontal treatment, are feasible and could influence clinical and inflammatory responses, particularly with respect to C‐reactive protein (CRP).

**Materials and Methods:**

Individuals with periodontitis were randomised to receive steps 1 and 2 of periodontal treatment, either following their regular diet (controls) or with three adjunctive 5‐day courses of FMD (test). Blood and gingival crevicular fluid (GCF) samples were collected to study the levels of inflammatory biomarkers, along with clinical parameters and patient‐reported outcome measurements (PROMs). All patients were followed up at days 1, 7, 45, 90 and 180 post treatment, and food diaries were completed. Exploratory repeated measures ANOVA and non‐parametric tests were used.

**Results:**

Twenty‐seven patients completed the 6‐month follow‐up. Feasibility criteria were satisfied. Baseline serum high‐sensitivity (hs)‐CRP increased on Day 1 in both groups and then decreased. Exploratory longitudinal analyses indicated temporal changes in hs‐CRP and selected GCF biomarkers (including MMP‐8, CRP, IL‐6, IL‐1*α* and IL‐1*β*), with descriptive trends towards lower values at later time points in the FMD group observed for hs‐CRP, MMP‐8 and IL‐6. No inter‐group periodontal clinical differences were detected. Only minimal adverse events were reported in the FMD group, with no inter‐group differences in PROMs and clinical outcomes.

**Conclusion:**

Three cycles of FMD, adjunctive to step 2, were associated with a reduction of the inflammatory response after subgingival instrumentation, although these changes did not benefit clinical parameters. Given the exploratory nature of the analyses and the multiple comparisons performed, these findings should be interpreted with caution.

## 1 Introduction

Although periodontitis is a microbially driven inflammatory disease that causes destruction of the tooth attachment apparatus, these inflammatory changes also manifest systemically, affecting other organs and tissues (Tonetti et al. 2018). Periodontal treatment, consisting of steps 1 and 2 (supra‐ and sub‐gingival instrumentation), is associated with a short‐term acute inflammatory response characterised by an increase in circulating acute‐phase inflammatory markers, followed by a longer term decrease in these biomarkers (Graziani et al. 2010). In fact, intervention trials have shown that intense treatment of periodontitis significantly reduces serum levels of biomarkers of systemic inflammation, such as C‐reactive protein (CRP) (D'Aiuto et al. 2018; Montero et al. 2020; Tonetti et al. 2007).

Although a moderate inflammatory response can be beneficial in the early stages of periodontal healing, chronic systemic inflammation can serve as a risk factor for various systemic diseases. Therefore, different strategies have been developed to reduce systemic inflammation in patients with periodontitis (Mainas et al. 2022; Neves et al. 2023). Periodic fasting lasting 2–5 days has proven to be beneficial for reducing biological age and lowering the risk of systemic diseases, while requiring only a 5‐day‐per‐month lifestyle change or less (Brandhorst et al. 2015, 2024). In particular, a novel approach called fasting‐mimicking diet (FMD) has shown very promising effects in reducing risk factors associated with ageing, diabetes, autoimmunity, cardiovascular disease, neurodegeneration and cancer (Longo et al. 2021). A recent systematic review suggested that caloric restriction may positively affect periodontal inflammation and treatment outcomes (Mainas et al. 2023) and, more specifically, recent studies on intermittent fasting reported promising results in terms of reduction of periodontal inflammation (Lira‐Junior et al. 2024; Pappe et al. 2025).

Recently, our group conducted a pilot trial in 20 subjects with severe periodontitis (stages III–IV). Participants were randomly assigned to receive step 2 periodontal therapy (subgingival instrumentation) with either one cycle of FMD or a regular diet and were followed for 3 months (Mainas et al. 2025). One cycle of FMD showed promising trends towards reductions of local and systemic inflammatory biomarkers but not in terms of clinical outcomes, when compared with the controls (Mainas et al. 2025).

Therefore, this study aimed to assess the effect of a three‐cycle FMD on clinical and systemic inflammation outcomes as an adjunct to steps 1 and 2 of periodontal therapy. The null hypothesis states that three cycles of the FMD diet combined with step 2 periodontal therapy do not affect CRP levels 90 days after treatment.

## 2 Materials and Methods

### 2.1 Study Population

This was a multi‐centre feasibility randomised controlled trial with an internal pilot. The CONSORT guidelines were followed (Hopewell et al. 2025). The study was registered on clinicaltrials.gov on 9 October 2023 (NCT06074861). Twenty‐eight suitable individuals with periodontitis referred for treatment were included in this study. Ethics approval for the analysis was obtained from the International University of Catalunya (PER‐ECL‐2022‐02), Complutense University of Madrid (23/411‐E), University of Santiago (2023/275), University of Granada (3530/CEIH/2023) and University of Murcia (578/2023). Each patient provided written consent to participate in the study. Patient visits took place from December 2023 to November 2024 at the periodontology departments of the five universities mentioned above.

Systemically healthy patients (18–70 years) with generalised periodontitis stage III–IV, grades B or C, (Papapanou et al. 2018), normal weight to overweight were included. Smokers and individuals who took medications and/or systemic antibiotic within 3 months preceding the study were excluded (more details regarding inclusion/exclusion criteria can be found in Supporting Information S1).

### 2.2 Randomisation and Allocation Concealment

Randomisation and allocation concealment were implemented using a computer‐generated random block design with a fixed block size of four, without stratification. Allocation concealment was achieved by using sealed envelopes, which were handled by personnel not directly involved in the study. Another investigator, who was not involved in the study, opened these envelopes during the baseline visit, communicated the allocation (test or control group) to patients and provided detailed instructions on the daily intake of products that individuals in the test group should consume, while controls were encouraged to continue with their usual diet. To further minimise potential sources of bias, both the examiner and the therapists were masked to the group assignment throughout the study. Masking was also preserved during the statistical analysis, and the randomisation code was broken only following analysis.

### 2.3 Clinical Examination

Baseline data included patient self‐reported medical and smoking histories. The following periodontal measurements were assessed through gentle probing with a UNC‐15 periodontal probe at six sites per tooth by one examiner at each centre: dichotomous full‐mouth plaque score (FMPS) (O'Leary et al. 1972), full‐mouth probing pocket depth (PPD), recession (REC) of the gingival margin from the cemento‐enamel junction (CEJ), bleeding on probing (BoP) (Ainamo and Bay 1975) and tooth mobility. Clinical attachment level (CAL) was calculated as the sum of PPD and REC. A Nabers probe was used to assess furcation involvement (Hamp et al. 1975).

All baseline and post‐treatment clinical measurements were performed by a single calibrated examiner at each centre. These examiners were blinded to the group allocation and completed a training exercise and intra‐examiner calibrations before commencing the study. This exercise consisted of measuring the outcome variable (PPD) in five subjects twice at six sites per tooth. Intra‐examiner calibration showed agreements within 1 and 2 mm PPD in 94.3% and 96.5% of cases, respectively. Inter‐examiner calibration was not feasible for practical reasons (see Table S2 for details concerning the intra‐examiner calibration).

### 2.4 Diet

**FIGURE 1:**
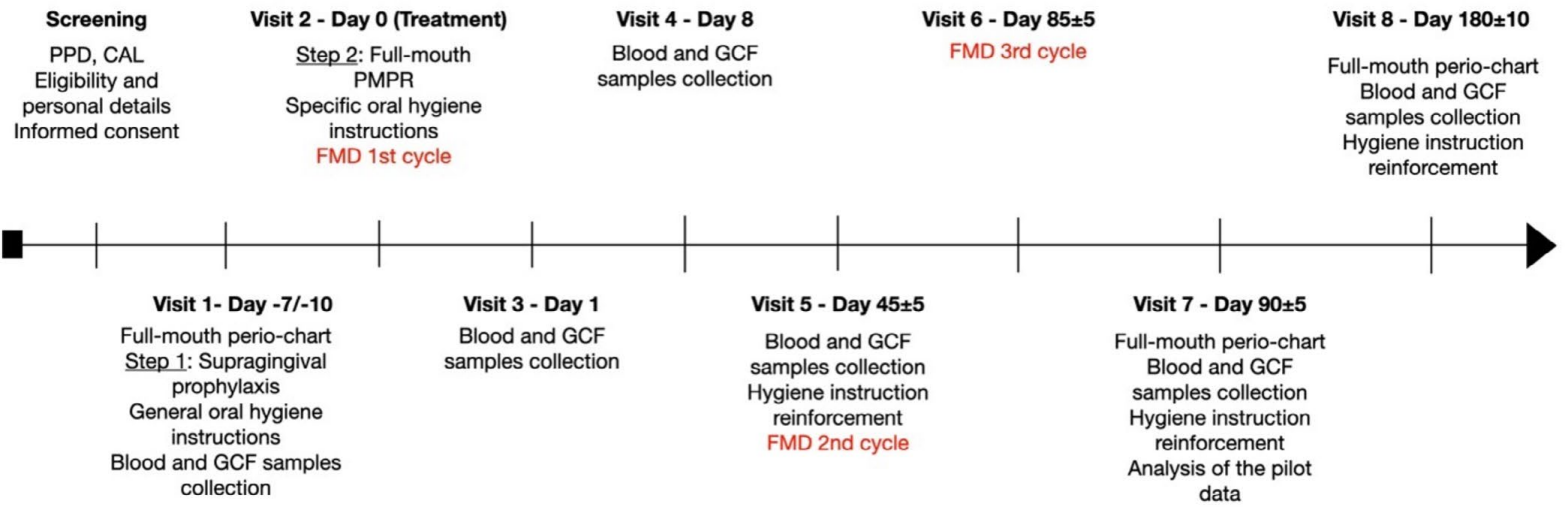
Flowchart of the study.

Patients from the test group completed three cycles of FMD (ProLon, L‐Nutra Inc., Los Angeles, CA, USA) (Mainas et al. 2025). The first cycle began on the same day as the scheduled subgingival instrumentation (specifically in the morning, at breakfast, before receiving the treatment). The second cycle was administered 45 days after the treatment, and the final cycle was given 85 days after the treatment (Figure 1). Details regarding the diet are reported in Supporting Information S3. The control group participants continued their usual (ad libitum) diet throughout the entire study.

Dietary questionnaires, such as food diaries, were collected from both the test and control groups at baseline and after 6 months to monitor food intake and assess participants' compliance.

### 2.5 GCF Sampling

In each study participant, gingival crevicular fluid (GCF) was collected from the mesial sulcus of each first molar using Periopaper (OraFlow Inc., New York, USA). Paper strips were inserted into the sulcus at the entrance until slight resistance was felt, then left in place for 30 s. Samples contaminated with blood or diluted with saliva during collection were discarded. GCF harvesting always preceded periodontal probing to avoid blood contamination. The strips were pooled in an Eppendorf tube and stored at −80°C. The samples were shipped to King's College London (UK) at the end of the study, where they were analysed (methodology related to GCF is detailed in Supporting Information S4).

### 2.6 Blood Sampling

Blood samples were collected from each participant using standard venepuncture techniques. Blood and serum (after centrifugation) were immediately aliquoted into 1.5‐mL Eppendorf tubes and stored at −80°C until analysis after transportation to King's College London (methodology related to blood is detailed in Supporting Information S5).

### 2.7 Biomarkers

The primary outcome of the study was the change in serum hs‐CRP levels between baseline and 90 days after treatment.

Serum and GCF samples were analysed according to the manufacturer's instructions, using high‐sensitivity multi‐analyte ELISAs (Ella Automated Immunoassay System; ProteinSimple) to measure levels of hs‐CRP in serum and interleukins 1 alpha (IL‐1*α*), IL‐1 beta (IL‐1*β*), IL‐6, IL‐10, CRP and MMP‐8 in GCF. Each sample was tested in duplicate for both procedures (see Supporting Information S6, where Ella technique is explained in detail).

### 2.8 Periodontal Treatment and Follow‐Ups

Participants received steps 1 and 2 of periodontal therapy in accordance with the clinical practice guidelines for treating periodontitis (Sanz et al. 2020), including oral hygiene instructions, risk factor management and supra‐ and sub‐gingival instrumentation. These steps were implemented simultaneously as part of a comprehensive full‐mouth treatment approach. Full‐mouth sub‐gingival instrumentation was administered in a single morning appointment using curettes and ultrasonic instruments under local anaesthesia. Then patients were scheduled for follow‐up examinations at 1, 90 and 180 days after treatment.

### 2.9 Patient‐Reported Outcome Measures (PROMs)

OHIP‐14 questionnaires were administered at baseline and at 6 months after treatment to evaluate patient‐reported outcomes on oral‐health‐related quality of life (PROMs) (Slade and Spencer 1994).

### 2.10 Statistical Analysis and Sample Size Calculation

This study was designed primarily to assess feasibility. All clinical and biomarker outcomes were considered exploratory and descriptive, and *p*‐values are reported for completeness without implying confirmatory evidence of treatment efficacy.

This trial included a convenience sample of 28 patients. Baseline characteristics were compared using independent *t*‐tests and *χ* ^2^ tests. Clinical parameters were analysed using paired and independent *t*‐tests. Longitudinal changes were explored using repeated‐measures ANOVA (time × group; Greenhouse–Geisser correction). Biomarker normality was assessed with the Shapiro–Wilk test, and between‐group comparisons were made using Wilcoxon tests. Spearman correlation assessed serum GCF CRP associations. Supporting Information S7 reports full details related to the statistics and feasibility/progression endpoints.

## 3 Results

**FIGURE 2:**
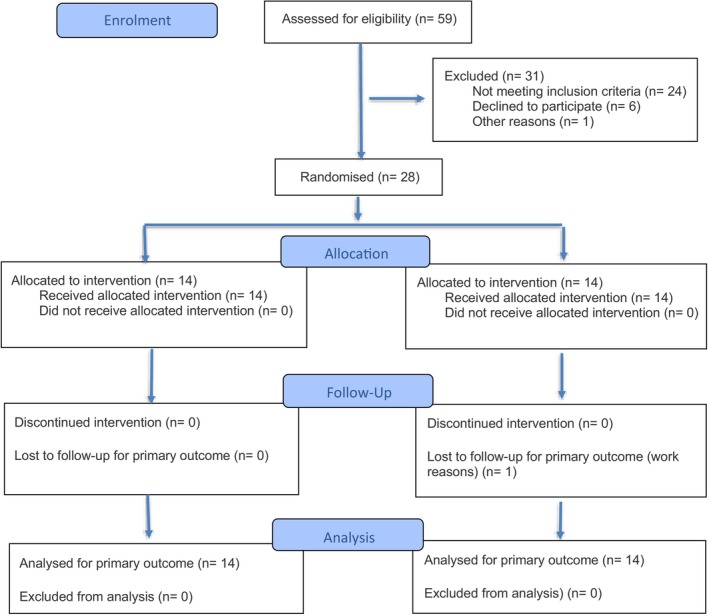
CONSORT 2025 flow diagram. Flow diagram of the progress through the phases of a randomised trial of two groups (that is, enrolment, intervention allocation, follow‐up, and data analysis).

A total of 28 patients participated in the study. Table 1 shows their baseline demographic and clinical characteristics. Subjects assigned to the test group had a higher percentage of females compared to the controls (86% vs. 50%), although these differences were not statistically significant. Also, there were no statistically significant differences for any of the measured clinical outcomes at baseline. Twenty‐seven participants completed all study visits up to the 6‐month visit. One subject in the control group was lost to follow‐up after changing jobs (Figure 2).

**TABLE 1:** Patient characteristics.

		Frequency/average mean (SD)	Frequency/average mean (SD)	Test vs. control *p‐*value
		Test (*n* = 14)	Control (*n* = 14)	
Age		48.2 (10.5)	53.3 (7.6)	0.248
BMI		24.96 (2.73)	25.1 (2.92)	0.70
Gender	Male	7	7	0.103
	Female	2	12	
Ethnicity	White	13	14	1.0
	Afro‐Caribbean	1	0	
Smoking	Never smokers	12	13	1.0
	Former smokers	2	1	
Periodontal diagnosis—Stage	III	9	7	0.704
	IV	5	7	
Periodontal diagnosis—Grade	B	11	10	1.0
	C	3	4	

*Note:* Mean and standard deviation are reported for continuous variables and frequency is reported for categorical variables.

Abbreviations: BMI, body mass index; SD, standard deviation.

### 3.1 Dietary Outcomes

All participants in the test group reported completing the three 5‐day FMD cycles, as confirmed by the food diary provided by the patients throughout the entire FMD cycle. These subjects did not report any serious adverse events, although five patients (36%) experienced minor side effects. In detail, one patient reported fatigue and headache on the first day of the first FMD cycle; one patient experienced nausea on the second day of the first FMD cycle; one patient reported fatigue and nausea on the first day of the second cycle; and two patients described episodes of fatigue and constipation during all cycles. All pre‐defined feasibility and progression criteria were fully met, with 100% adherence based on participant food diaries, as reported in Supporting Information.

No significant changes in dietary patterns were observed from the start to the end of the study, although some minor changes, such as decreased red meat intake and increased fish and vegetable consumption, were noted in about 85% of FMD patients only (see Supporting Information S8 for an in‐depth analysis concerning the food diaries).

### 3.2 Clinical Outcomes

The total time spent on subgingival instrumentation was 78 min in the test group and 80 min in the control group. Table 2 shows the clinical parameters of both groups at baseline, 3 and 6 months. Within each group, significant changes in all clinical parameters were observed from baseline to 3 months and from baseline to 6 months. However, no differences were found between the groups at any time point.

**TABLE 2:** Clinical characteristics of test and control groups at baseline and post‐step 2 periodontal therapy.

	Baseline		3 months post step 2 periodontal therapy		6 months post step 2 periodontal therapy		Overall difference baseline‐ 3 months *p*‐value	Overall difference baseline‐ 6 months *p‐*value	Inter‐group difference baseline‐ 3 months *p*‐value	Inter‐group difference baseline‐ 6 months *p*‐value
	Test	Control	Test	Control	Test	Control				
Full‐mouth plaque score	60.0 (27.7)	66.5 (53.5)	35.1 (29.0)	30.4 (23.9)	21.1 (14.9)	22.7 (13.7)	< 0.001	< 0.001	0.649	0.779
Full‐mouth bleeding score	52 (19.8)	58.2 (21.3)	25.7 (16.4)	35.0 (16.4)	20.3 (12.4)	22.3 (13.9)	< 0.001	< 0.001	0.239	0.687
Average PPD (mm)	3.25 (0.78)	3.48 (0.79)	2.86 (0.72)	2.80 (0.71)	2.7 (0.64)	2.83 (0.56)	< 0.001	< 0.001	0.757	0.929
Average CAL (mm)	3.61 (1.01)	4.0 (1.07)	3.4 (1.17)	3.51 (0.75)	3.35 (1.05)	3.60 (0.92)	0.018	0.005	0.962	0.643
Number of PPDs > 4 mm	29.5 (27.0)	33.9 (30.4)	18.1 (17.3)	18.1 (18.9)	14.6 (15.0)	16.0 (12.6)	0.002	< 0.001	0.998	0.801
Number of PPDs > 5 mm	15.9 (17.3)	20.7 (25.1)	8.6 (9.3)	7.9 (10.7)	7.6 (10.8)	6.6 (8.0)	0.004	< 0.001	0.855	0.751

*Note:* Means and standard deviations are reported.

Abbreviations: CAL, clinical attachment level; PPD, probing pocket depth.

‘Pocket closure’ (reduction of PPD to < 5 mm) was attained in 27% and 30% of cases at 6 months for the control and test groups, respectively. Endpoints of therapy (no PPD ≥ 6 mm and no PPD > 4 mm with no BoP) were reached in seven test and seven control patients (50% of the sample).

Given the number of biomarkers and time point comparisons analysed, these statistically significant findings should be interpreted cautiously because of the increased risk of type I error.

### 3.3 Serum hs‐CRP

**FIGURE 3:**
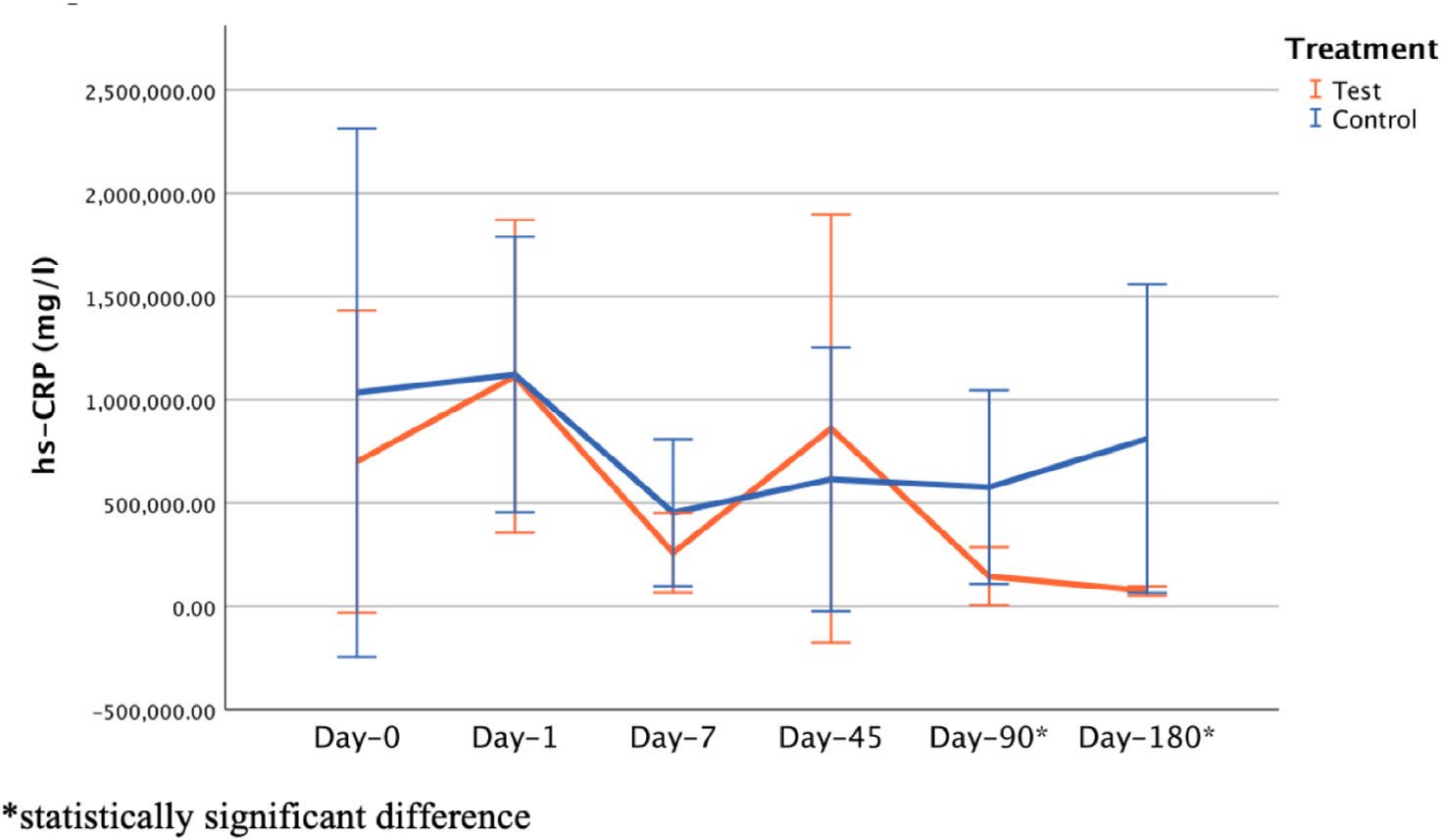
Line graph illustrating serum hs‐CRP levels in test and control patients at all study timepoints.

Table 3 and Figure 3 display the changes in serum hs‐CRP levels from baseline to 6 months for patients by treatment group.

**TABLE 3:** Serum hs‐CRP levels in test and control patients at all study timepoints, reported as median and interquartile range.

		Test (*n* = 14)	Control (*n* = 14)	*p*‐value for difference test‐control
Average hs‐CRP (mg/L)	Day 0	0.95 (2.35)	2.36 (1.62)	0.520
	Day 1	4.62 (5.0)	5.34 (4.71)	0.528
	Day 7	0.90 (1.84)	3.29 (1.53)	0.073
	Day 45	0.83 (1.92)	2.27 (2.37)	0.497
	Day 90	0.18 (0.21)	2.18 (2.69)	0.003
	Day 180	0.21 (0.19)	2.35 (2.98)	0.008
Repeated‐measures ANOVA (Greenhouse–Geisser corrected): – Time effect: *p* = 0.026. – Group × Time interaction: *p* = 0.039				

Abbreviation: hs‐CRP, high‐sensitivity C‐reactive protein.

hs‐CRP levels were higher in the control group than in the test group at baseline (not statistically significant). Exploratory analysis showed that median hs‐CRP at baseline was 0.95 mg/L in the FMD group and 2.30 mg/L in the control group, increasing at Day 1 to approximately 4.60 and 5.30 mg/L, respectively (*p* < 0.001), then decreasing to less than 0.90 mg/L at Day 7 and Day 45 in the FMD group but remaining at 3.20 and 2.20 mg/L in the control group at days 7 and 45, respectively (inter‐group differences not significant). Repeated‐measures ANOVA revealed a significant main effect of time on hs‐CRP levels (Greenhouse–Geisser corrected *p* = 0.026), indicating changes in hs‐CRP over the follow‐up period. The group × time interaction reached statistical significance (*p* = 0.039).

### 3.4 Biomarkers in GCF

**FIGURE 4:**
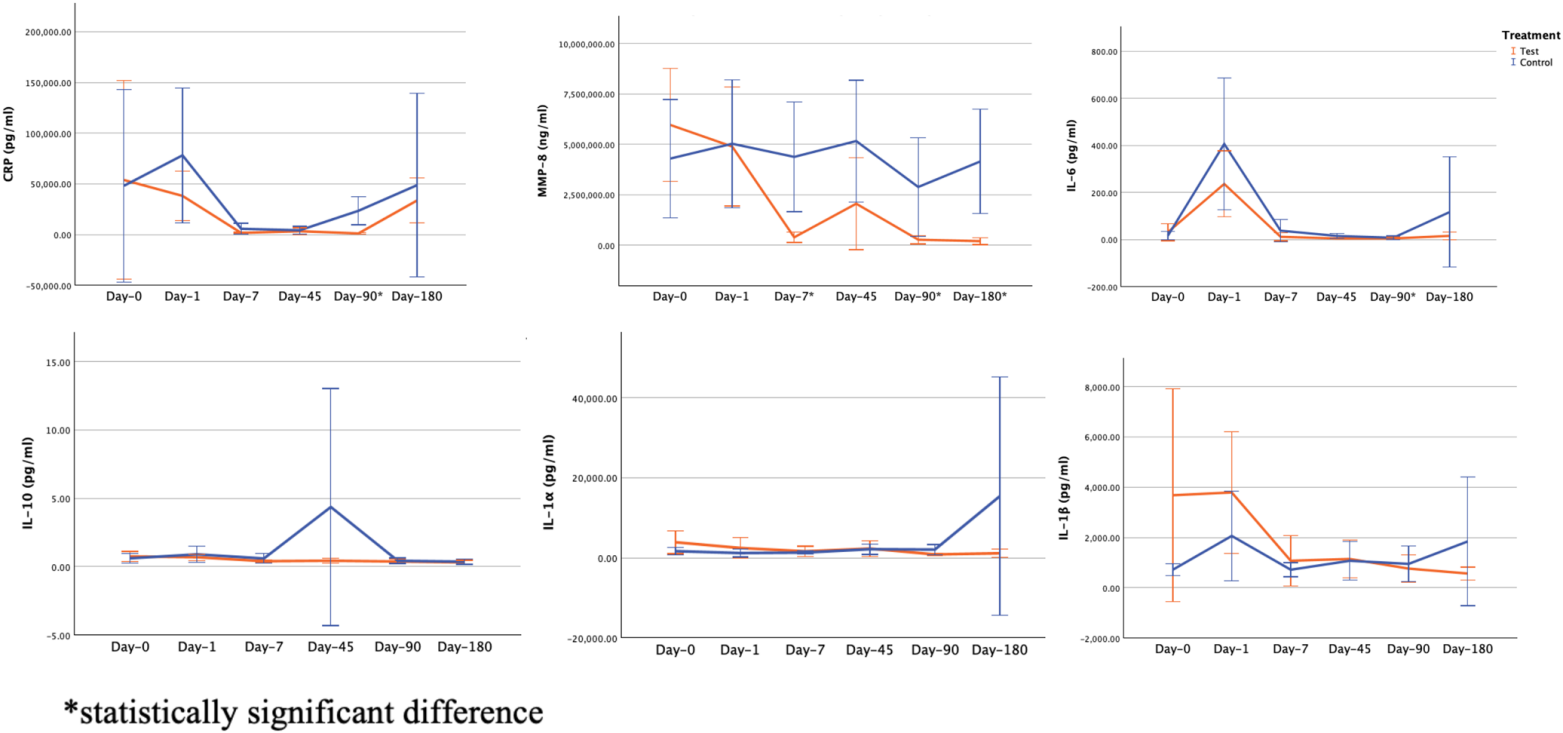
Line graphs of GCF markers levels in test and control patients at all study timepoints.

Figure 4 illustrates the changes in GCF biomarker levels over the six study time points in patients grouped by treatment. Exploratory analysis found that most biomarkers tended to decrease from baseline to Day 180 in the test group, while in the control group there was an increase at Day 1, then a decrease up to Day 90 and an increase again until Day 180. Exploratory repeated‐measures ANOVA revealed significant main effects of time for CRP, MMP‐8 and IL‐6 (all Greenhouse–Geisser corrected *p* < 0.05), indicating temporal changes following non‐surgical periodontal therapy. No statistically significant group × time interactions were observed for CRP, IL‐10, IL‐1*α* or IL‐1*β*. A significant group × time interaction was observed for MMP‐8 (*p* = 0.013) and IL‐6 (*p* = 0.045) (more details regarding the GCF levels of biomarkers are in Table S9).

Bivariate correlation analysis showed significant correlations at all time points between serum and GCF levels of CRP (Table S10 gives the complete information).

### 3.5 PROMs

No significant differences between groups were observed for the total OHIP‐14 score, but only the test group showed a significant reduction in the total score after 6 months (*p* = 0.030) (full details concerning PROMs are provided in Supporting Information S11).

## 4 Discussion

This study demonstrated the safety and feasibility of three cycles of FMD as an adjunct to periodontal subgingival instrumentation, as evidenced by complete self‐reported adherence to the three 5‐day diet cycles and fulfilment of the progression criteria. The decision to administer three cycles of FMD at approximately monthly intervals was based on established clinical protocols used in previous studies on metabolic and inflammatory conditions. Prior human trials have demonstrated that periodic 5‐day FMD cycles repeated every 3–4 weeks are sufficient to induce sustained and cumulative metabolic and inflammatory adaptations while maintaining safety and acceptability (Brandhorst et al. 2015; Kulkarni et al. 2026; Wei et al. 2017). The decision to start the first FMD cycle on the same day as sub‐gingival instrumentation was primarily pragmatic, intended to standardise intervention timing and optimise adherence within the clinical workflow, as no evidence currently defines the optimal timing of fasting‐based interventions relative to periodontal therapy. Later sampling points were also intended to explore potential cumulative inflammatory effects of repeated FMD cycles, particularly after the second and third cycle, rather than the short‐term response to a single cycle.

The feasibility of FMD was confirmed as all 14 test participants who completed the regimen, aligning with existing scientific evidence in other contexts (Wei et al. 2017). Only minor adverse events were reported, indicating that FMD may be suitable for use in normal‐weight, systemically healthy patients with periodontitis because, when used as an adjunct to non‐surgical periodontal treatment, it apparently modulated both local and systemic inflammation.

Interestingly, in patients in the test group, dietary habits shifted slightly after completing the study, with a reduction in the intake of meats, cakes and sweets, as well as an increase in the consumption of fish, fresh fruit, vegetables and legumes. Similar findings have been reported in another study on FMD (Brandhorst et al. 2015).

Systemic hs‐CRP levels and a panel of six biomarkers measured in GCF were examined in both test and control group patients before and after periodontal therapy using a comprehensive full‐mouth disinfection approach, as reported in a previous investigation evaluating the impact of treatment on systemic acute‐phase responses (Graziani et al. 2015). In the test group using FMD, the serum hs‐CRP levels after periodontal treatment aligned with existing literature, showing an increase immediately after sub‐gingival instrumentation (Day 1), followed by a decrease from Day 7 to Day 180 (Machado et al. 2021). However, in the control group, hs‐CRP levels returned to baseline levels after the 7 days and remained stable throughout the 6 months. This pattern has also been reported in other studies after subgingival instrumentation (Boduroglu et al. 2017; D'Aiuto et al. 2006; Kamil et al. 2011). It should also be considered that, in small feasibility samples with relatively low baseline inflammatory levels, apparent reductions in hs‐CRP may partly reflect inter‐individual biological variability or statistical floor effects rather than a definitive treatment‐induced systemic modulation. CRP is considered a robust marker of systemic inflammation, found to be slightly elevated in patients with periodontitis (D'Aiuto et al. 2004) and to decrease following effective periodontal treatment (Paraskevas et al. 2008), indicating a sustained systemic response when these improvements are maintained over 6 months (Van Dyke and Kornman 2008).

The plausibility of these reductions in the group with three cycles of FMD could potentially be explained by prior studies in other clinical models suggesting that FMDs may influence systemic inflammation through pathways involving IGF‐1 signalling, ketogenesis, oxidative stress reduction and autophagy (Damas et al. 2025; de Cabo and Mattson 2019; Michalsen and Li 2013; Parveen 2021). However, these pathways were not directly assessed here and have mainly been reported in other medical fields. Hence, they remain speculative in the context of periodontitis, as the present study does not provide mechanistic proofs and the null hypothesis cannot be formally rejected. Serum CRP levels can be influenced by numerous systemic or environmental factors beyond periodontal status, including transient, subclinical or asymptomatic infections, minor injuries and hormonal or metabolic fluctuations (Mendelsohn et al. 2025). Although participants were systemically healthy and followed a standardised protocol, unmeasured inflammatory events over the 6‐month period cannot be fully excluded (Mendelsohn et al. 2025).

The effect of both treatment regimens was also tested at the local level by evaluating target cytokines in GCF. Exploratory repeated ANOVA revealed trends favouring the test group in terms of reduction of levels of MMP‐8, which may be relevant since MMP‐8 is a collagenase that plays a key role in the breakdown of periodontal connective tissue during the initiation and progression of periodontitis (Sorsa et al. 2006). Similarly, levels of IL‐6 in GCF were significantly lower in the FMD group 3 months post treatment, which corroborates the impact of FMD on the local inflammatory response since IL‐6 is a pro‐inflammatory cytokine involved in immune regulation and inflammatory processes (Neurath and Kesting 2024), and elevated IL‐6 concentrations in GCF have been shown to correlate with periodontal pocket depth and disease severity (Lin et al. 2005).

Despite differences in both local and systemic inflammation biomarkers, the clinical outcomes after periodontal therapy showed no significant differences between the groups, indicating that the FMD regimen used was unable to influence the patient's response following therapy. This lack of effect of FMD on clinical parameters may have been limited by the short follow‐up period, sample size, baseline disease severity or numerous confounders impacting the results of periodontal therapy (Polimeni et al. 2006; Preshaw et al. 2004).

Although the present intervention differs in modality and timing, existing human studies suggest that fasting‐based dietary approaches may be associated with reductions in periodontal inflammatory parameters (Lira‐Junior et al. 2024; Pappe et al. 2025). In a 6‐month pilot study of overweight individuals following a 5:2 intermittent fasting regimen, significant reductions in BoP and GCF volume were observed without concomitant changes in plaque levels (Lira‐Junior et al. 2024). Similarly, during experimentally induced gingivitis, both time‐restricted eating (16:8) and religious dry fasting were associated with attenuated increases in BoP and GCF compared with a regular diet, despite comparable plaque accumulation (Pappe et al. 2025).

The results from this trial, however, should be considered with caution considering the existing critical limitations, which include the study design (feasibility), the small sample size, the use of multiple examiners/operators and lack of inter‐examiner calibration, the lack of objective biochemical markers of fasting compliance (such as IGF‐1 or *β*‐hydroxybutyrate level (ketons) elevation, or changes in fasting glucose/insulin ratios), which limit the precision of adherence assessment. Furthermore, compliance was based only on self‐reported food diaries, and metabolic parameters were not collected, limiting the assessment of systemic metabolic responses to FMD. Other limitations include lack of power to detect clinical efficacy and the short‐term follow‐up, the baseline imbalance observed for GCF IL‐1*β* levels between groups, which may influence the interpretation of longitudinal comparisons for this specific biomarker, and the imbalance between groups in the proportion of male and female patients.

## 5 Conclusions

Within its limitations, this pilot study showed the feasibility of three cycles of an FMD as an adjunct to periodontal treatment in patients with periodontitis stages III–IV. The tested intervention resulted in changes in local and systemic inflammatory responses; however, these findings should be interpreted as exploratory. Minor side effects with no differences between groups in clinical parameters were detected. Exploratory biomarker trends indicate that further investigation in a fully powered trial is warranted, although no clinical efficacy conclusions can be drawn at this stage.

## Author Contributions

G.M., M.I., M.V. and L.N. contributed to the conception and design of the study. G.M. and L.N. coordinated the trial and drafted the manuscript. E.F., M.A.B., J.D., F.J.G.M, A.M‐F., I.C., G.P.Z., J.G.S., J.N., A.S.A. and C.P.C. contributed to the clinical phases of the study and collected the data. G.M. performed the microbiological assessments. G.M. and L.N. interpreted the data and performed the statistical analysis. All authors made substantial contributions to this study, and critically reviewed and revised the manuscript.

## Funding

This study was funded by a Medical Research Council‐Impact Accelerator Account (MRC‐IAA) grant. L‐Nutra Inc. provided the kits of the diet (ProLon) in kind.

## Conflicts of Interest

V.D.L. declares that he has equity interest in L‐Nutra Inc., the company that produces the fasting‐mimicking diet and holds patents related to the FMD. However, he does not receive consulting from the company and all of his patent royalties have been assigned to charitable organisations. All laboratory analyses and statistical evaluations were performed independently of L‐Nutra Inc., which had no role in study protocol development, in data handling or interpretation or in manuscript preparation. The remaining authors declare no conflicts of interest in connection with this article.

## Supporting information


**Supporting Information: S1.** Inclusion and exclusion criteria in full.
**Supporting Information: S2.** Details of intra‐examiner calibration for each centre.
**Supporting Information: S3.** Details on fasting‐mimicking diet (FMD) – ProLon.
**Supporting Information: S4.** Details of GCF samples collection and storage.
**Supporting Information: S5.** Details of blood samples collection and storage.
**Supporting Information: S6.** Details of Ella procedure.
**Supporting Information: S7.** Full details of statistical analysis and sample size calculation, including feasibility aspects and progression criteria.
**Supporting Information: S8.** Full details of dietary habits recorded on questionnaires.
**Supporting Information: S9.** Tables of GCF markers levels in test and control patients at all study timepoints, reported as median and interquartile ranges.
**Supporting Information: S10.** Table of bivariate correlation between levels of CRP in serum and GCF.
**Supporting Information: S11.** Full details of PROMs.

## Data Availability

The data that support the findings of this study are available from the corresponding author upon reasonable request.
