# Supplementary material for: A Fasting‐Mimicking Diet Affects the Inflammatory Response Following Periodontal Treatment: A Multi‐centre Feasibility Randomised Controlled Pilot Trial
Source: J Clin Periodontol. 2026 Jun 10;53(8):1254–64. doi: 10.1111/jcpe.70139 (PMC13371414; doi:10.1111/jcpe.70139)
Supplement: Supplementary file 1 — Supporting Information: S1. Inclusion and exclusion criteria in full. Supporting Information: S2. Details of intra‐examiner calibration for each centre. Supporting Information: S3. Details on fasting‐mimicking diet (FMD) – ProLon. Supporting Information: S4. Details of GCF samples collection and storage. Supporting Information: S5. Details of blood samples collection and storage. Supporting Information: S6. Details of Ella procedure. Supporting Information: S7. Full details of statistical analysis and sample size calculation, including feasibility aspects and progression criteria. Supporting Information: S8. Full details of dietary habits recorded on questionnaires. Supporting Information: S9. Tables of GCF markers levels in test and control patients at all study timepoints, reported as median and interquartile ranges. Supporting Information: S10. Table of bivariate correlation between levels of CRP in serum and GCF. Supporting Information: S11. Full details of PROMs. [file JCPE-53-1254-s001.docx]

**Supportive information**

**S1. Inclusion and exclusion criteria in full.**

Eligible patients were recruited if they fulfilled the following criteria:

- subjects between 18-70 years with a minimum of 24 teeth present.

- Diagnosis of Periodontitis, stages III-IV generalized, grades B or C, (Papapanou et al., 2018).

- Self-reported systemic health

- Body mass index: 18.5-30 kg/m^2^ (normal weight to overweight, but not obese)

- Willing and competent (verbally and cognitively) to provide written informed consent and complete a medical history form

- Willing and able to perform all study procedures.

Exclusion Criteria were:

- Periodontal treatment in the last 12 months

- Presence of hopeless teeth, acute dental conditions, teeth with endodontic-periodontal lesions, and necrotizing periodontal diseases

- Smoking (self-reported use of any cigarettes or electronic cigarettes for at least 5 years)

- Mental illness, including severe depression

- Drug dependency

- Hormone replacement therapy (Dehydroepiandrosterone (DHEA), oestrogen, thyroid, testosterone)

- Severe hypertension (systolic blood pressure (BP) > 200 mm Hg and/or diastolic BP > 105 mm Hg).

- Currently taking part in other clinical trial

- Pregnant or breastfeeding women

- Taking medications and/or systemic antibiotic intake within 3 months preceding the study

- Current orthodontic treatment

- Alcohol intake greater than two drinks daily for women and three drinks daily for men

- Denture wearer/presence of dental implants

- Untreated caries and other significant oral diseases

- Unable or unwilling to complete the dietary intervention

- Special dietary requirements incompatible with the study interventions

- Significant food allergies

**S2. Intra-examiner calibration for each centre.**

| **Centre** | **Agreement within 1 mm (%)** | **Agreement within 2 mm (%)** |
| --- | --- | --- |
| Barcelona | 91.8% | 95.2% |
| Madrid | 96.5% | 97.8% |
| Santiago | 92.4% | 94.7% |
| Granada | 95.7% | 97.2% |
| Murcia | 94.9% | 96.5% |
| **Overall** | **94.3%** | **96.5%** |

**S3. Details on fasting-mimicking diet (FMD) - ProLon**

The FMD program is a plant-based diet designed to mimic fasting effects while providing both macro- and micronutrients to reduce the burden and adverse effects of fasting (Wei et al., 2017). The FMD regime included ingredients that are generally recognized as safe (GRAS) and consists of vegetable-based soups, energy bars, energy drinks, cracker snacks, olives, herbal teas, and supplements. All items were individually packaged for daily use. Water (recommended at about 2 litres per day) was the only item participants could consume from outside the package during the diet cycle. The FMD involved a 5-day regimen that provided approximately 1100 calories on the first day and about 750 calories each day from the second to the fifth day (Wei et al., 2017). On the 6^th^ day, known as the transition day, patients gradually resumed their usual food habits, with a return to a regular diet by the 7^th^ day.

To ensure consistent implementation of the fasting-mimicking diet protocol across centres, each site designated a trained investigator responsible for patient guidance throughout all FMD cycles. These investigators, who also handled allocation concealment, received standardized instruction from a registered nutritionist with extensive expertise in FMD clinical protocols. They provided detailed verbal and written guidance at baseline and maintained regular phone contact with participants during each diet cycle, serving as the point of reference for any questions or concerns.

**S4. GCF samples**

Gingival crevicular fluid (GCF) was collected using Periopaper (OraFlow Inc., New York, USA) from the mesial sulcus of the first molars of each study participant. GCF sampling was performed prior to periodontal probing, to avoid blood contamination. Periopaper was placed at the entrance of the sulcus until slight resistance was felt and then left for 30s. Samples visually contaminated with blood or diluted with saliva during sampling were discarded. The strips were pooled in an Eppendorf tube and transferred to − 80 ◦C. Periopaper samples were not normalized to total protein concentration, as biomarker quantification was performed on pooled GCF eluates collected under standardized sampling conditions. The samples were sent to King’s College London (United Kingdom) at the end of the study, where GCF was extracted using PBS with protease inhibitors 1X (1 tablet for 10 ml of PBS) (Complete ULTRA tablets, Mini; EDTA-free). Briefly, 25 μl of PBS/protease inhibitor cocktail was added and centrifuged at 11,000 g for 15 min at 4 ◦C. An additional 25 μl of PBS/protease inhibitor cocktail was then added and centrifuged at 11,000 g for 15 min at 4 ◦C, to a total volume of 50 μl. The eluted GCF was stored at – 80°C until analysis.

**S5. Blood samples**

Blood samples were collected from each participant using standard venepuncture techniques. Blood samples consisted of two tubes: one for blood and one for serum. A total of 13–15 mL of whole blood was drawn into sterile vacutainer tubes containing clot activator and gel separator. The tubes were gently inverted to ensure proper mixing of the anticoagulant and the blood sample. Blood was immediately aliquoted in Eppendorfs and stored at − 80 ◦C. Subsequently, the other samples were allowed to clot at room temperature for 30 min to one hour to facilitate serum separation. Following clot formation, the tubes were centrifuged at 4000 rpm for 5 min. This centrifugation step effectively separated the serum from the clot and cellular components. The resulting serum samples were then carefully transferred into labelled microcentrifuge tubes using a sterile pipette and stored at – 80°C until analysis following transportation to King’s College London.

**S6. Ella procedure**

The Ella platform (Ella™ Automated Immonoassay System; ProteinSimple) was used to perform multiplex analysis of serum inflammatory biomarkers, allowing simultaneous quantification of multiple analytes in a single run with high sensitivity and reproducibility. This automated immunoassay system utilizes a microfluidic cartridge pre-loaded with specific capture antibodies for each analyte of interest. Serum samples were thawed, centrifuged to remove any debris, and loaded into the cartridge at the recommended dilution factor to ensure accurate detection within the dynamic range of the assay. Each sample was tested in triplicate. The Simple Plex system automatically handled all pipetting, washing, and incubation steps. Following the immunoassay, the cartridge was analysed by the Ella reader, which measured the fluorescence intensity of the detection antibodies bound to each analyte. The results were automatically generated as analyte concentrations (pg/mL) using built-in software that calculates values based on pre-defined standard curves. The use of Ella minimized variability and reduced processing time compared to traditional ELISA methods, making it an ideal tool for high-throughput biomarker analysis. The system's precision and accuracy were verified through quality control checks before each assay run.

**S7. Statistical analysis and sample size calculation**

This study was designed primarily to assess feasibility aspects. All clinical and biomarker outcomes were considered exploratory and descriptive. P-values are reported for completeness but should not be interpreted as confirmatory evidence of treatment efficacy.

In the absence of preliminary data in the literature, this study was designed as a randomized controlled trial with an internal pilot, including analysis after a convenience sample of 28 patients, to determine if more patients were needed to achieve sufficient power to test the main study hypothesis and to inform the design and sample size estimation of future adequately powered confirmatory studies.

Feasibility was evaluated using specific ‘progression criteria’. Patient characteristics were evaluated using independent sample t-tests for continuous variables and Chi-square tests for categorical variables. Continuous variables are presented as means and standard deviations, while categorical variables are presented as frequencies.

Clinical periodontal parameters, including PPD, CAL, FMPS, and FMBS, were calculated for each patient by averaging site-level measurements across the entire dentition. Patient-level means were then derived and used as the unit of analysis for statistical comparisons. For the number of sites with PPD > 4 mm and > 5 mm, the total count of sites meeting these thresholds was determined per patient and used for group-level comparisons. The paired t-test and independent sample t-test were used to evaluate significant changes in clinical parameters between baseline and 3 months, and between baseline and 6 months for both groups combined and between groups, respectively. Repeated-measures ANOVA was employed to identify significant changes in clinical parameters over time, comparing baseline, 3 months, and 6 months, with the patient serving as the unit of analysis.

The Shapiro-Wilk Test was used to determine if the studied biomarkers followed a normal distribution. Since no normal distribution was found, non-parametric tests (Wilcoxon) were employed for inter-group hs-CRP and all other biomarker analyses to detect differences at each time point. The Friedman test was applied to analyse differences in biomarkers across all time points for both groups combined. Longitudinal changes in systemic and local inflammatory biomarkers were explored using repeated-measures ANOVA, with time (six timepoints) as the within-subject factor and allocation group as the between-subject factor. Mauchly’s test was used to assess sphericity, and Greenhouse–Geisser correction was applied when this assumption was violated. Spearman’s rank correlation coefficient was calculated to assess the correlation between serum and GCF CRP levels.

**Feasibility aspects and progression criteria.**

| **FEASIBILITY ASPECTS** | **PROGRESSION CRITERIA** | **ACHIEVED OR NOT** |
| --- | --- | --- |
| Willingness to be randomised | ≥ 50% of approached patients agree | YES |
| Self-reported compliance with diet | ≥ 66% of test group patients self-report compliance (defined as not more than 1 day in which the diet was not followed) | YES |
| Acceptance of blood samples | ≥ 66% of recruited patients have all blood samples taken as per protocol | YES |
| Absence of serious adverse events | No patients report serious adverse events which might be related to the test diet | YES |

**S8. Dietary habits**

At baseline, only one patient from the test group reported to be vegetarian. Two test and one control patient reported to be on a weight-loss diet. Two test and one control participants reported to be on a hypocaloric diet. One test individual was following a sugar-free and low-fat diet. Among the other controls, one was on an intermittent fasting, one followed a soft diet, another one a ketogenic diet, one was on a cutting and bulking diet, and one was on a gluten-free and lactose-free diet.

Overall, all patients reported a varied food intake at baseline, including regular intake of eggs, milk, cheese/dairy products, whole grains, and lean proteins, with some patients reporting higher red and processed meat consumption than others.

At the end of the study, around 85% of participants (12 out of 14) belonging to the test group showed slight changes in their diet. In details, 3 patients decreased their weekly read meat consumption, 2 patients decreased their weekly process meat consumption, 2 patients decreased cakes/sweets weekly intake, 7 patients increased their weekly fish consumption, 3 patients increased their legumes weekly intake and 3 patients eliminated the intake of carbonated drinks. Besides, 6 patients increased the daily portion consumption of fresh fruit and 5 patients increased the daily portion intake of vegetables.

Furthermore, 5 patients showed a slight increase in daily water consumption.

No substantial changes were noted in terms of weekly consumption of whole grains, nuts, milk and cheese/dairy products. In contrast, patients from the control group did not show any changes in their dietary habits.

**S9. GCF markers levels in test and control patients at all study timepoints, reported as median and interquartile ranges.**

Exploratory analyses of local inflammatory biomarkers are presented below and should be interpreted descriptively.

|  |  | **Test**  **(n=14)** | **Control (n=14)** | ***p* value for difference test-control** | |
| --- | --- | --- | --- | --- | --- |
| **Average MMP-8**  **(mg/l)** | Day 0 | 4.85 (6.33) | 2.90 (6.51) | 0.097 |  |
|  | Day 1 | 2.59 (6.15) | 4.65 (4.66) | 0.959 |  |
|  | Day 7 | 2.76 (4.59) | 4.49 (3.94) | 0.002 |  |
|  | Day 45 | 1.10 (4.53) | 4.36 (4.35) | 0.062 |  |
|  | Day 90 | 0.22 (0.40) | 1.97 (6.67) | 0.009 |  |
|  | Day 180 | 0.19 (0.42) | 4.31 (4.82) | <0.001 |  |
| Repeated-measures ANOVA (Greenhouse–Geisser corrected):  – Time effect: *p*=0.011  – Group × Time interaction: *p*=0.013 | | | | |  |
| **Average CRP**  **(mg/l)** | Day 0 | 0.59 (0.01) | 0.51 (0.26) | 0.662 |  |
|  | Day 1 | 0.36 (0.58) | 0.81 (1.58) | 0.662 |  |
|  | Day 7 | 0.01 (0.001) | 0.06 (0.11) | 0.174 |  |
|  | Day 45 | 0.003 (0.07) | 0.012 (0.08) | 0.626 |  |
|  | Day 90 | 0.007 (0.01) | 0.08 (0.07) | 0.023 |  |
|  | Day 180 | 0.28 (0.001) | 0.53 (0.12) | 0.094 |  |
| Repeated-measures ANOVA (Greenhouse–Geisser corrected):  – Time effect: *p*=0.026  – Group × Time interaction: *p*=0.075 | | | | |  |
| **Average**  **IL-6**  **(pg/ml)** | Day 0 | 15.85 (50.67) | 14.89 (41.64) | 0.145 |  |
|  | Day 1 | 206.4 (395.6) | 330.1 (631.9) | 0.190 |  |
|  | Day 7 | 11.51 (42.25) | 41.42 (123.9) | 0.275 |  |
|  | Day 45 | 4.30 (7.20) | 12.37 (18.53) | 0.786 |  |
|  | Day 90 | 0.91 (1.50) | 4.74 (7.15) | 0.033 |  |
|  | Day 180 | 2.24 (3.17) | 7.38 (21.10) | 0.099 |  |
| Repeated-measures ANOVA (Greenhouse–Geisser corrected):  – Time effect: *p<*0.001  – Group × Time interaction: *p*=0.045 | | | | |  |
| **Average**  **IL-1α**  **(pg/ml)** | Day 0 | 3852 (5459) | 1347 (1143) | 0.771 |  |
|  | Day 1 | 2045 (3460) | 1165 (1482) | 0.560 |  |
|  | Day 7 | 1432 (1784) | 1252 (747) | 0.072 |  |
|  | Day 45 | 1928 (2730) | 2117 (1862) | 0.124 |  |
|  | Day 90 | 858 (727) | 1922 (2063) | 0.015 |  |
|  | Day 180 | 1080 (1538) | 14661 (47000) | 0.409 |  |
| Repeated-measures ANOVA (Greenhouse–Geisser corrected):  – Time effect: *p*=0.350  – Group × Time interaction: *p*=0.276 | | | | |  |
| **Average**  **IL-1β**  **(pg/ml)** | Day 0 | 3802 (11441) | 737 (547) | 0.017 |  |
|  | Day 1 | 3499 (5804) | 1894 (4397) | 0.159 |  |
|  | Day 7 | 1291 (2773) | 701 (684) | 0.560 |  |
|  | Day 45 | 1132 (2080) | 1113 (1931) | 0.913 |  |
|  | Day 90 | 775 (1305) | 1012 (1987) | 0.846 |  |
|  | Day 180 | 552 (845) | 1664 (5980) | 0.264 |  |
| Repeated-measures ANOVA (Greenhouse–Geisser corrected):  – Time effect: *p*=0.111  – Group × Time interaction: *p*=0.149 | | | | |  |
| **Average**  **IL-10**  **(pg/ml)** | Day 0 | 0.64 (0.67) | 0.57 (0.74) | 0.467 |  |
|  | Day 1 | 0.67 (0.55) | 0.88 (1.39) | 0.808 |  |
|  | Day 7 | 0.41 (0.23) | 0.63 (0.82) | 0.771 |  |
|  | Day 45 | 0.44 (0.42) | 4.29 (18.73) | 0.828 |  |
|  | Day 90 | 0.35 (0.31) | 0.43 (0.44) | 0.627 |  |
|  | Day 180 | 0.28 (0.38) | 0.36 (0.43) | 0.467 |  |
| Repeated-measures ANOVA (Greenhouse–Geisser corrected):  – Time effect: *p*=0.333  – Group × Time interaction: *p*=0.335 | | | | |  |

MMP-8: Matrix Metalloproteinases-8; CRP: C Reactive Protein; IL-6: Interleukin-6; IL-1α: Interleukin-1 alfa; IL-1β: Interleukin-1 beta; IL-10: Interleukin-10

**S10. Bivariate correlation between levels of CRP in serum and GCF.**

|  |  | **CRP**  **(Day-0)** | **CRP**  **(Day-1)** | **CRP**  **(Day-7)** | **CRP**  **(Day-45)** | **CRP**  **(Day-90)** | **CRP**  **(Day-180)** |
| --- | --- | --- | --- | --- | --- | --- | --- |
| **hs-CRP**  **(Day-0)** | **Correlation Coefficient** | **0.832*** |  |  |  |  |  |
|  | ***p* value** | **0.045** |  |  |  |  |  |
| **hs-CRP**  **(Day-1)** | **Correlation Coefficient** |  | **0.742**** |  |  |  |  |
|  | ***p* value** |  | **<0.001** |  |  |  |  |
| **hs-CRP**  **(Day-7)** | **Correlation Coefficient** |  |  | **0.731**** |  |  |  |
|  | ***p* value** |  |  | **<0.001** |  |  |  |
| **hs-CRP**  **(Day-45)** | **Correlation Coefficient** |  |  |  | **0.628**** |  |  |
|  | ***p* value** |  |  |  | **<0.001** |  |  |
| **hs-CRP**  **(Day-90)** | **Correlation Coefficient** |  |  |  |  | **0,551**** |  |
|  | ***p* value** |  |  |  |  | **0.003** |  |
| **hs-CRP**  **(Day-180)** | **Correlation Coefficient** |  |  |  |  |  | **0.709**** |
|  | ***p* value** |  |  |  |  |  | **<0.001** |

****p*<0.005; ***p*<0.001**

**S11. PROMs**

No difference between groups at Day-0 (*p*=0.60) and at Day-180 (*p*=0.90).

Significant difference intragroup for the Test group (*p*=0.030) but not for the Control group (*p*=0.581).

Overall difference between groups were not statistically significant (*p*=0.181).
